# The Role of Sleep for Age-Related Differences in Neurobehavioral Performance

**DOI:** 10.3390/life14040496

**Published:** 2024-04-11

**Authors:** Orna Tzischinsky, Efrat Barel

**Affiliations:** 1 Department of Behavioral Sciences and the Center for Psychobiological Research, The Max Stern Academic College of Emek Yezreel, Emek Yezreel 1930600, Israel; 2 School of Psychological Sciences, University of Haifa, Haifa 3103301, Israel; ebarel@staff.haifa.ac.il

**Keywords:** sleep, neurobehavioral performance, children, adolescents, young adults, PVT, DSST

## Abstract

This study investigated developmental changes from childhood to adulthood in neurobehavioral performance and sleep measures. While many studies have examined age-related changes between childhood and adolescence and from mid-to-late adulthood, young adulthood has been overlooked. The main aim of this study was to investigate the effects of sleep loss on developmental changes in neurobehavioral performance and sleepiness in a natural setting. A total of 119 children, adolescents, and young adults (38 children aged 6–9; 38 adolescents aged 13–19; and 43 young adults aged 20–27) wore an actigraph for a continuous five-weekday night. Subjective sleepiness (Karolinska Sleepiness Scale) and neurobehavioral performance (using the psychomotor vigilance test and the digit symbol substitution test) were measured on five school days. The results showed that adolescents and young adults outperformed children on both the digit symbol substitution test and the psychomotor vigilance test measures. However, adolescents committed more errors of commission on the psychomotor vigilance test and reported higher levels of subjective sleepiness. The results are discussed in relation to brain maturation in various cognitive functions.

## 1. Introduction

Sleep is vital for human health and is necessary for optimal functioning across life domains. Numerous studies have demonstrated that sleep impacts neurocognitive performance [1], including attentional processing [2], executive functioning [3], learning and memory [4], and emotional regulation [5]. Changes in sleep quantity and quality across development reflect brain maturation that impacts cognition [6]. The relationship between sleep and neurobehavioral performance among children, adolescents, and young adults has not been explored in detail.

Sleep is controlled by various factors, including biological and environmental factors. Developmental changes throughout life affect sleep patterns with age differences in sleep onset [7], sleep duration [8], and sleepiness [9,10,11]. During childhood, the sleep schedule on schooldays and weekends is generally constant [12]. When children enter adolescence, their sleep patterns change drastically, with a decrease in sleep length that can mainly be explained by the shift of sleep onset time to later hours, early wake-up time on weekdays, and increased sleep length during weekends and vacations [13]. The decrease in sleep time has also been demonstrated across the transition to adulthood [14,15].

Apart from age-related changes in sleep patterns, studies have investigated the developmental course of various cognitive domains. While general intelligence measures have been reported as relatively stable across development [16], age-related changes have been documented for executive domains, especially attention and memory [17,18]. Gur and colleagues [19] employed a computerized neurocognitive battery in a population of youths aged 8–21. They found substantial improvement with age for executive control functions, especially attention and motor speed. These domains have been linked to prefrontal lobe function, where maturation is delayed relative to other brain regions [20,21,22].

Sustained attention is critical for everyday functioning that bolsters learning, memory, executive functioning, and other cognitive domains [23,24]. It connects to the ability to maintain attention and task management over time [25]. Age-related changes in sustained attention have demonstrated improved performance from childhood to adolescence [26]. However, studies examining age-related changes in sustained attention performance from adolescence to adulthood have demonstrated inconsistent findings: some have shown moderated improvement that peaks in the mid-40s (among 10–70-year-olds) [27]; some have shown no difference in performance between younger adults (18–32-year-olds) and older adults (60–74-year-olds) [28]; and others have shown improved performance in older adults (55–77-year-olds) [29]. These inconsistencies may be attributed to research design characteristics, such as the type and form of measurement. In their review, Ferguson and colleagues [30] investigated age-related differences in executive functioning. They found that inhibitory control and planning were higher in younger adults than in adolescents. Functional magnetic resonance imaging (fMRI) studies investigated the neural correlates of the altered performance in sustained attention from childhood to adulthood. Increased activation in the right inferior frontal and temporoparietal regions during sustained attention tasks was found to be positively associated with increasing age [31]. Support for this evidence emerged in several other studies, especially among children and adolescents, highlighting these periods in development in which important changes occur in neural substrates associated with functional developmental changes [32,33,34]. For example, Casey and colleagues suggested that the observed changes in impulsivity and risk-taking behaviors across development are accompanied by changes in functional connectivity in the limbic corticosubcortical circuitry [32]. A meta-analysis including fMRI studies revealed a quadratic developmental trajectory within the frontoparietal network [33]. Another complex developmental trajectory was found in a structural MRI study. In a large cohort of 375 participants (3.5–33-year-olds), Shaw and colleagues [34] showed a cubic developmental trajectory in cortical thickness, with initial childhood increase followed by adolescent decline and then stabilization of cortical thickness in young adulthood.

Studies investigating the effect of sleep on cognitive performance revealed that sleep has a differential impact on cognitive performance across the lifespan. A meta-analysis showed that the magnitude of the effect size increased with age, from a small negative effect observed in school-aged children and adolescents to a moderate-to-large negative effect in adults [35]. Many studies addressed the effects of sleep loss on cognitive domains in young adults and found that sleep deprivation results in increases in reaction time and lapses [36,37]. Sustained attention is most susceptible to sleep loss and may be the fundamental factor that explains much of the variance in other cognitive impairments following sleep loss [38]. Among the few studies addressing sleep and attention in childhood and adolescence, Spruyt and colleagues [39] found that across development, various sleep parameters, such as sleep duration, sleep regulation, and sleep midpoint, become increasingly important for attentional performance.

In addition to age-related changes in sleep patterns and attentional performance, with the transition from childhood to adolescence, there is an increase in subjective sleepiness [9,10,11]. Only a few studies have addressed age-related changes in attention and sleepiness. Campbell and colleagues [40] found an improvement in attention performance alongside an increase in sleepiness with age. Based on the synaptic elimination model [39], they posited that elevated brain activity in adolescence increases the need for the sleep-dependent recuperative process and that a restriction of sleep increases sleepiness. Synaptic elimination, on the other hand, reduces the redundant functional circuits, which may explain the improvement in attention performance.

Previous studies examining the role of sleep loss on attention performance used various measures of attention properties. Among the most widely used measures are the psychomotor vigilance test (PVT) and the digit symbol substitution test (DSST). In various studies, sleep was shown to be associated with PVT and DSST performance; indeed, sleep loss was found to lead to slower response time, increased lapses of attention, and false starts on the PVT [41,42,43] and to fewer correct responses on the DSST [2,44].

Given these developmental changes in sleep architecture (i.e., sleep patterns such as sleep stages, sleep onset-time and wake-time, and sleep duration) as well as in cognitive performance, we conducted a cross-sectional study to examine developmental changes in neurobehavioral functioning. By conducting this study in the natural setting of the home, we were able to follow the age-related changes in sleep patterns from childhood to adulthood and examine developmental changes in neurobehavioral functioning in connection with sleep. Many studies have investigated age-related changes in neurobehavioral functioning from childhood to adolescence and from middle to late adulthood; however, young adulthood has been overlooked when examining lifespan changes. Therefore, this study compared three age groups using the same tasks for all participants in the same natural setting. We predicted that adolescents and young adults would outperform children in neurobehavioral functioning and that sleep efficiency would be associated with neurobehavioral functioning, especially in children and adolescents. Finally, based on the synaptic elimination model [39], we hypothesized that adolescents would report higher levels of sleepiness than children and young adults.

## 2. Materials and Methods

### 2.1. Participants

This study comprised 119 children, adolescents, and young adults, divided into three groups: 38 children—22 females (mean age 7.45 ± 0.91 years, ranging 6–9 years) and 16 males (mean age 7.50 ± 0.52 years, ranging 7–8 years), before puberty according to their parents’ reports; 38 adolescents—21 females (mean age 16.14 ± 2.01 years, ranging 13–19 years) and 17 males (mean age 16.41 ± 1.97 years, ranging 13–19 years), after puberty according to their parents’ reports; and 43 adult students—22 females (mean age 22.36 ± 1.47 years, ranging 20–25 years) and 21 males (mean age 24.05 ± 1.66 years, ranging 21–27 years). Inclusion criteria included females and males ranging from 6–27 years; physically and psychologically healthy; no ADHD; no clinically significant abnormalities; and no history of psychiatric illness.

There was no statistically significant difference in gender distribution between the 3 groups (*p* = 0.83). Each group’s data were composed distinctly. For each group, the age range was selected according to the association between sleep and puberty [45].

### 2.2. Materials

#### 2.2.1. Cognitive Performance Measures

The participants completed two cognitive tests.

**A visual Psychomotor Vigilance Test** (PVT-B; Joggle Research Program, Seattle, WA, USA). The PVT-B is a three-minute-long sustained attention that was performed on an iPad. The subjects were asked to keep vigilant attention on a target box and to reply rapidly to the appearance of a stimulus while avoiding untimely answers. The PVT is employed as a sensitive test of sustained attention performance under short sleep duration or variations in circadian phases [46,47,48]. The outcome measures were mean reaction time (RT), false starts, and lapses. Lapses are defined as ≥500 ms latency between stimulus presentation and subject response.

**Digit Symbol Substitution Test (DSST)**. The DSST was performed on an iPad and included the matching of digits (1–9) to symbols [49]. It is a subject-paced task, and the number of accurate responses in 60 s was used as a measure of cognitive processing speed. The DSST is a subtest of the Wechsler Adult Intelligence Scale–Third Edition (WAIS-III) and has respectable validity and reliability (test–retest = 0.83; reliability coefficient = 0.93) [50]. The consequence measures of the DSST are right replies and the mean RT.

#### 2.2.2. Sleep Measures

An actigraph is a small device worn on the wrist of the non-dominant hand for five consecutive weeknights to assess sleep patterns (Mini-Act, AMA-32, AMI). Data are collected (in 1 min epochs) and analyzed by a computer using the ActionW software (Version 2, using Sadeh algorithm #20). with a validated algorithm [51,52,53]. The actigraph was used to measure sleep onset time, wake time, sleep efficiency ([total sleep time/total time in bed] × 100), sleep duration, wake after sleep onset (WASO: the number of minutes during the sleep period scored as awake), and sleep latency.

Subjective Sleepiness: We also used a Karolinska Sleepiness Scale (KSS) questionnaire [54]. The KSS is a ruler containing nine statements connected to sleepiness. Scores vary from 1 (extremely alert) to 9 (extremely sleepy), with higher scores pointing to greater personal sleepiness. The KSS is commonly used among a range of age groups, among them children and adolescents [55], and has demonstrated a correlation with objective drowsiness, e.g., [56]. The subjects completed the KSS questionnaire in the morning after wake-up, in the afternoon after school between 2 and 5 PM, and before bedtime. The scores were averaged per day.

### 2.3. Statistical Methods

Normality and homogeneity of variances were assessed using the Shapiro–Wilk test (normality) and Levene’s test (homogeneity of variance) for each variable in each age group. For non-parametric variables, a Kruskal–Wallis test was performed to test group differences in objective and subjective sleep variables and for each of the cognitive performance measures. Spearman correlations were performed to assess the association between sleep and performance by age group. All analyses were performed using SPSS (version 28). Significance was set at *p* < 0.05.

### 2.4. Power Analysis

A power analysis using G*Power 3.1.9.7 [57] indicated that 32 participants per group would be necessary to achieve 80% power and an alpha error rate of 0.05 with the expectation of detecting a medium effect size.

## 3. Results

### 3.1. Sleep

Table 1 presents the objective and subjective sleep data by group. There were statistically significant differences between the three groups in objective sleep. Post hoc testing revealed that children went to bed significantly earlier than both adolescents and young adults and that adolescents went to bed significantly earlier than young adults. Young adults woke up significantly later than both adolescents and children. Children had significantly longer sleep duration than both adolescents and young adults. In addition, adolescents had significantly lower sleep efficiency than both children and young adults. Adolescents had significantly longer WASO than both children and young adults, while young adults had significantly longer WASO than children. Adolescents had significantly higher KSS than both children and young adults.

### 3.2. Neurobehavioral Performance

As expected, there was a statistically significant difference in DSST performance measures between the three age groups (see Table 2). Post hoc testing revealed that children had fewer correct responses and slower reaction times than adolescents and young adults.

Furthermore, there was a statistically significant difference in PVT performance measures between the three age groups. Children had a greater number of lapses, slower reaction times, and fewer responses than adolescents and young adults. On the other hand, adolescents had a greater number of false starts than children.

### 3.3. Sleep and Neurobehavioral Performance

Subjective sleep (KSS) was not correlated with objective sleep (sleep efficiency). Within each age group, DSST was not significantly correlated with the sleep measures (objective and subjective). Among children, PVT reaction time and lapses were negatively correlated with sleep efficiency: the higher the sleep efficiency, the lower the reaction time and lapses. Among adolescents, PVT false starts were negatively correlated with sleep efficiency: the higher the sleep efficiency, the fewer the false starts. For the subjective sleep measure, positive correlations were found in adolescents between KSS and PVT reaction time and lapses: the more the adolescents reported sleepiness, the longer the reaction time and the greater the lapses (see Table 3). However, no significant correlation coefficients were found after applying Bonferroni corrections for multiple comparisons.

## 4. Discussion

This study explored developmental differences in neurobehavioral performance from childhood to young adulthood. DSST and PVT performance differed by age group, with adolescents and young adults outperforming children in all measures except for false starts. Children showed slower reaction time and fewer correct responses as measured by the DSST compared to adolescents and young adults. The DSST is classified as a measure of complex executive functioning, which includes various cognitive components, such as visual discrimination/identification, information processing speed, and manipulation of information [58,59]. Our findings are consistent with earlier reports of significant effects of age on DSST performance [44,60]. For example, Wu and colleagues [44] examined age-related differences in cognitive functioning, including DSST. They found that adolescents outperformed children, demonstrating fewer lapses and answering more items correctly. A few studies examined the developmental trajectory of DSST performance between adolescence and young adulthood and provided inconsistent findings. For example, Sudarshan and colleagues [61] showed that DSST peaks later in development. They assessed the cognitive performance of participants aged 16 to 69 using the Wechsler Adult Intelligence Scale and found that digit symbol coding peaks at the age of 23 and then declines progressively. Hartshorne and Germine [62] compared the age of peak performance across cognitive domains among 48,537 participants. They found considerable heterogeneity regarding the age at which cognitive abilities peak, with digit symbol coding peaking earlier (late adolescence) than other abilities that show continued improvement past early adulthood. In concordance with their findings, our findings suggest that DSST performance peaks in adolescence and is maintained throughout young adulthood. The search for the unique developmental pattern of each cognitive ability is manifested in the research of life span trajectories of crystallized (e.g., vocabulary) versus fluid intelligence (e.g., working memory, processing speed). Fluid intelligence refers to the ability to solve novel, abstract problems and does not depend on task-specific knowledge [63,64]; crystallized intelligence, on the other hand, is defined as acculturation knowledge [65]. In contrast to crystallized intelligence, which continues to improve across most of the life span and declines only in very old age, fluid intelligence is marked by an early rise and decline [66]. Neuroimaging studies provide support for the age-related decline in fluid intelligence. For example, Kalbitzer [67] investigated healthy participants aged 22–61 using PET and radioligand FDOPA to measure age-related differences in measures of fluid intelligence. They showed that cognitive speed (measured by the DSST) was associated with the magnitude of the prefrontal cortex (PFC) to synthesize catecholamines; both had a direct negative correlation with age.

As expected, children also had a greater number of lapses and slower reaction times than adolescents and young adults in PVT measures (except for false starts). These findings are in line with previous studies showing rapid improvement in sustained attention throughout childhood and adolescence [27]. Our findings are consistent with previous studies demonstrating improvement in PVT performance from childhood to adolescence (e.g., [68]). However, in line with the findings related to DSST performance, PVT performance too peaked in adolescence and remained stable through young adulthood. In a large study with 5325 children and adults, RTs in PVT were fastest among individuals in their 20s, with a decline in performance throughout adulthood [69]. Functional neuroimaging studies of pediatric populations demonstrated that increased activation in mainly right-lateralized brain areas during sustained attention tasks was positively correlated with increasing age in children and adolescents [70]. As an executive control process, attention is supported by the frontal lobes. It has been suggested that cognitive processes supported by the prefrontal cortex would manifest decline at an earlier age than other cognitive processes supported by non-frontal regions [71]. Indeed, studies demonstrated that executive performance, including processing speed and sustained attention, showed a substantial decline beginning in early adulthood [72].

Although adolescents exhibited equivalent performance to young adults and both groups outperformed children, they showed significantly higher PVT errors of commission (false starts) in comparison to both children and young adults. Errors of commission suggest a lack of inhibitory control [73] and increased impulsivity [74]. Inhibitory control is one of the basic mechanisms of executive functioning [75]. Previous studies suggested that executive functioning should be considered a multi-component construct in which each component develops differently and reaches maturation at different ages [76,77]. Indeed, neuroimaging studies provide support for the continuing maturation throughout adolescence of prefrontal connectivity with brain areas that require top-down processes for optimal and sustained inhibitory control [78]. While the connectivity of prefrontal systems undergoes significant maturation in childhood and reaches adult levels by adolescence, prefrontal connections with other cortical and subcortical regions continue to mature and strengthen through adolescence [79]. This gradual maturation in inhibitory control may explain this study’s results, with adolescents outperforming children in reaction time and number of correct responses while underperforming in errors of commission.

This study further aimed to investigate the association between sleep efficiency and neurobehavioral performance. We found that among children, PVT reaction time and lapses were negatively correlated with sleep efficiency: that is, the higher the sleep efficiency, the lower the reaction time and lapses. No correlation between sleep efficiency and PVT reaction time and lapses was found for adolescents and young adults. However, among adolescents, the PVT number of errors of commission was negatively correlated with sleep efficiency: the higher the sleep efficiency, the fewer the false starts. Previous studies exploring the relationship between sleep measures and PVT performance produced inconsistent findings [80]. Several studies compared this relationship between children and adolescents. Spruyt and colleagues [39] examined the role of sleep in the attentional performance of children and adolescents aged 6–18 in a natural setting. They demonstrated that sleep is imperative to attentional performance in a differential manner for children and adolescents: while in children, sleep is important for speed in attentional performance, in adolescence, sleep is important for both speed and stability of attention. In this study, adolescents had significantly lower sleep efficiency and exhibited higher levels of false starts than both children and young adults. Furthermore, a negative correlation between sleep efficiency and false starts was only found among adolescents. Previous studies showed that poor sleep quality is associated with less effortful control of attention among adolescents (e.g., [81,82]). In an fMRI study, Telzer and colleagues [83] showed that poor sleep quality was related to dampening activation in the dorsolateral prefrontal cortex (DLPFC) during response inhibition in adolescents. The lateral PFC is involved in cognitive and impulse control [21] and was shown in greater recruitment during risk-taking among adults compared to adolescents [84]. Telzer and colleagues suggested that dampening DLPFC activation following poor sleep may imply a relatively immature use of this region.

Adolescents demonstrated increased subjective sleepiness compared to children and young adults. Furthermore, positive associations between sleepiness and PVT mean RT and lapses were only found among adolescents: that is, higher levels of perceived sleepiness were associated with worse performance on PVT measures (but not with errors of commission). However, adolescents’ performance was still better than children’s. These paradoxical results are in accordance with Campbell and colleagues’ [29] findings about increases in both daytime subjective sleepiness and daytime sustained attention among adolescents. Campbell and colleagues [40] interpreted their results in relation to the model of adolescent brain development by synaptic elimination [85]. In recent examinations of the brain–sleep structure relationship across development, an association was found between the decrease in gray matter thickness and a decline in slow-wave electroencephalogram in adolescence [86,87]. Ong and colleagues [88] found that age-related improvement in processing speed among adolescents was associated with reduced gray matter thickness as well as reduced sleep slow wave activity and was not affected by sleepiness levels. The synaptic elimination model posits that in adolescence, as synaptic pruning proceeds, the intensity of brain activity declines, leading to increased sleepiness. At the same time, synaptic elimination reduces redundant neuronal pathways, which contributes to improvements in response efficiency and processing speed [40].

Despite its important contributions, this study has some limitations. First, by being conducted in a natural setting, it is not, in essence, so experimental. Given the reduced internal validity of such a setting, findings should be interpreted carefully. Nevertheless, the use of the same tasks in the same natural setting across all three groups provided an opportunity to directly investigate age-related differences in neurobehavioral performance. Furthermore, the use of natural settings enables an imitation of the “real world” effects of partial sleep deprivation, which thus strengthens external and ecological validity.

Second, we included limited measures of neurobehavioral performance across three age groups. We suggest that future studies expand the age range and cognitive domains in order to uncover the age of peak across cognitive domains. Understanding the differential trajectory in which each cognitive domain develops and peaks will facilitate laying the foundation to develop directed interventions addressing age-related cognitive decline.

Finally, our findings are restricted to the short-term outcomes of sleep loss on DSST and PVT performance. Future studies should examine the long-term outcomes of sleep loss on both brain structure and function across development.

In sum, this study investigated age-related differences in neurobehavioral performance. Adolescents and young adults outperformed children in DSST and PVT measures (except for error of commission) in spite of elevated subjective sleepiness reported by adolescents. It also investigated the effects of objective sleep measures on neurobehavioral performance in each age group. Sleep efficiency (objectively measured) was associated with PVT reaction time and lapses among children, whereas it was associated with errors of commission among adolescents. These developmental patterns were supported by neuroimaging studies showing age-dependent functional networks.

## 5. Conclusions

Our results suggest that age is associated with neurobehavioral performance, showing that increasing age is associated with better performance on DSST and PVT measures that reach a peak in adolescence and remain stable in young adulthood. Furthermore, although exhibiting high performance, adolescents showed higher levels of error of commission, suggesting an ongoing process of brain development related to control inhibition that will reach maturation in adulthood. Finally, objective and subjective sleep measures were highly influential for the adolescent group. Their neurobehavioral performance was impacted by sleep loss and sleepiness in sustained attention measures. Therefore, given the imperative role of sustained attention in general cognitive functioning and in everyday tasks, it is important to establish recommendations for sleep duration for adolescents.

## Figures and Tables

**Table 1:** Sleep data by group.

	Children	Adolescent	Young Adults	Kruskal–Wallis *χ*^2^		*p*
Objective sleep						
Onset	21.48 ± 0.63 [20.27–22.80]	23.76 ± 1.08 [22.10–26.80]	24.82 ± 1.19 [22.15–27.63]	75.62.		<0.001
Wake	6.84 ± 0.47 [5.87–8.10]	7.04 ± 0.80 [5.92–10.17]	8.00 ± 1.24 [4.70–10.98]	27.06		<0.001
Duration	562.3 ± 44.4 [445.0–629.2]	444.8 ± 40.7 [371.8–515.6]	430.0 ± 59.5 [298.2–534.0]	61.31		<0.001
Sleep efficiency (%)	95.2 ± 3.3 [87.7–100.0]	84.9 ± 4.4 [75.6–92.7]	95.8 ± 2.9 [88.1–99.3]	65.41		<0.001
WASO (min)	27.4 ± 18.3 [1.6–65.6]	40.7 ± 12.2 [14.2–73.0]	17.5 ± 12.5 [2.7–51.0]	34.87		<0.001
Subjective sleep						
KSS	4.2 ± 2.0 [1–8]	5.6 ± 1.6 [3–8]	4.4 ± 1.9 [1–9]	10.66		0.005

Note: WASO—wake after sleep onset; KSS—the Karolinska Sleepiness Scale.

**Table 2:** Neurobehavioral data by group.

	Children (*n* = 38)	Adolescents (*n* = 38)	Young Adults (*n* = 43)	Kruskal–Wallis *χ*^2^	*p*
DSST					
Correct Responses	56.5 ± 12.6 [30–82]	81.3 ± 10.1 [53–106]	83.4 ± 10.6 [43–98]	58.68	<0.001
Mean RT	1444.5 ± 310.8 [954.0–2192.3]	985.5 ± 150.8 [723.8–1515.7]	953.6 ± 110.5 [767.4–1341.5]	60.57	<0.001
PVT					
Lapses	13.3 ± 7.7 [1–26]	5.8 ± 4.9 [0–23]	5.4 ± 5.9 [0–25]	28.09	<0.001
False Starts	0.67 ± 0.89 [0–3]	2.81 ± 1.72 [0–7]	0.33 ± 0.52 [0–2]	30.56	<0.001
Mean RT	398.2 ± 113.2 [250.9–696.2]	277.8 ± 45.9 [201.0–390.0]	253.1 ± 47.5 [184.4–444.7]	51.88	<0.001

Note: DSST—Digit Symbol Substitution Test; Mean RT—mean reaction time; PVT—Psychomotor Vigilance Test.

**Table 3:** Spearman correlations between objective and subjective sleep measures and neurobehavioral performance.

	KSS	DSST		PVT		
Sleep Measures		Correct Responses	Reaction Time	Lapses	False Starts	Mean RT
Sleep Efficiency						
Children	−0.02	0.19	−0.13	−0.30 *	−0.07	−0.29 *
Adolescents	−0.07	0.01	−0.01	−0.01	−0.44 *	−0.10
Young Adults	−0.12	−0.12	0.12	0.15	0.12	0.18
KSS						
Children		−0.01	−0.05	−0.16	0.18	0.21
Adolescents		0.01	−0.01	0.41 *	−0.08	0.42 *
Young Adults		−0.17	0.21	0.09	−0.13	0.18

Note: DSST—Digit Symbol Substitution Test; Mean RT—mean reaction time; PVT—Psychomotor Vigilance test. * *p* < 0.05 (one-tailed).

## Data Availability

The data that support the findings of this study are available from the corresponding author upon request.
